# Supplementary material for: Structure of SRSF1 RRM1 bound to RNA reveals an unexpected bimodal mode of interaction and explains its involvement in SMN1 exon7 splicing
Source: Nat Commun. 2021 Jan 18;12:428. doi: 10.1038/s41467-020-20481-w (PMC7813835; doi:10.1038/s41467-020-20481-w)
Supplement: Supplementary file 1 — Supplementary Information [file 41467_2020_20481_MOESM1_ESM.pdf]

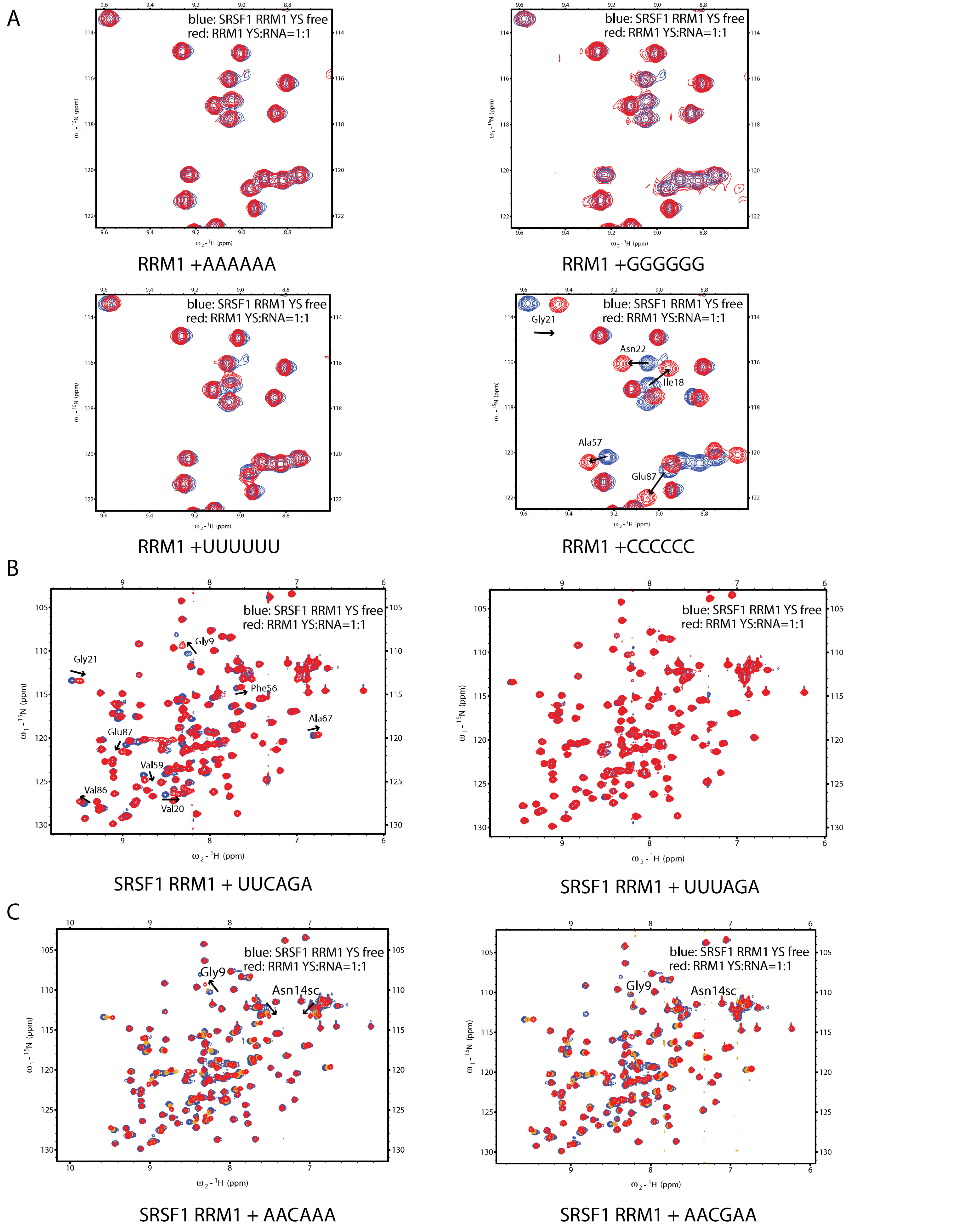

Figure S1: SRSF1 RRM1 binds preferentially to cytosines
(A) Overlay of 1H-15N HSQC spectra measured with SRSF1 RRM1 free form (in blue) and in the presence of polyA, polyG, polyC or polyU molecules (in red). **(B)** Overlay of 1H-15N HSQC spectra measured with SRSF1 RRM1 free form (in blue) and in the presence of SMN1 ESE1 (UUCAGA) or SMN2 ESE1 (UUUAGA) (in red). **(C)** Overlay of 1H-15N HSQC spectra measured with SRSF1 RRM1 free form (in blue) and in the presence of AACAAA or AACGAA RNA (in red).

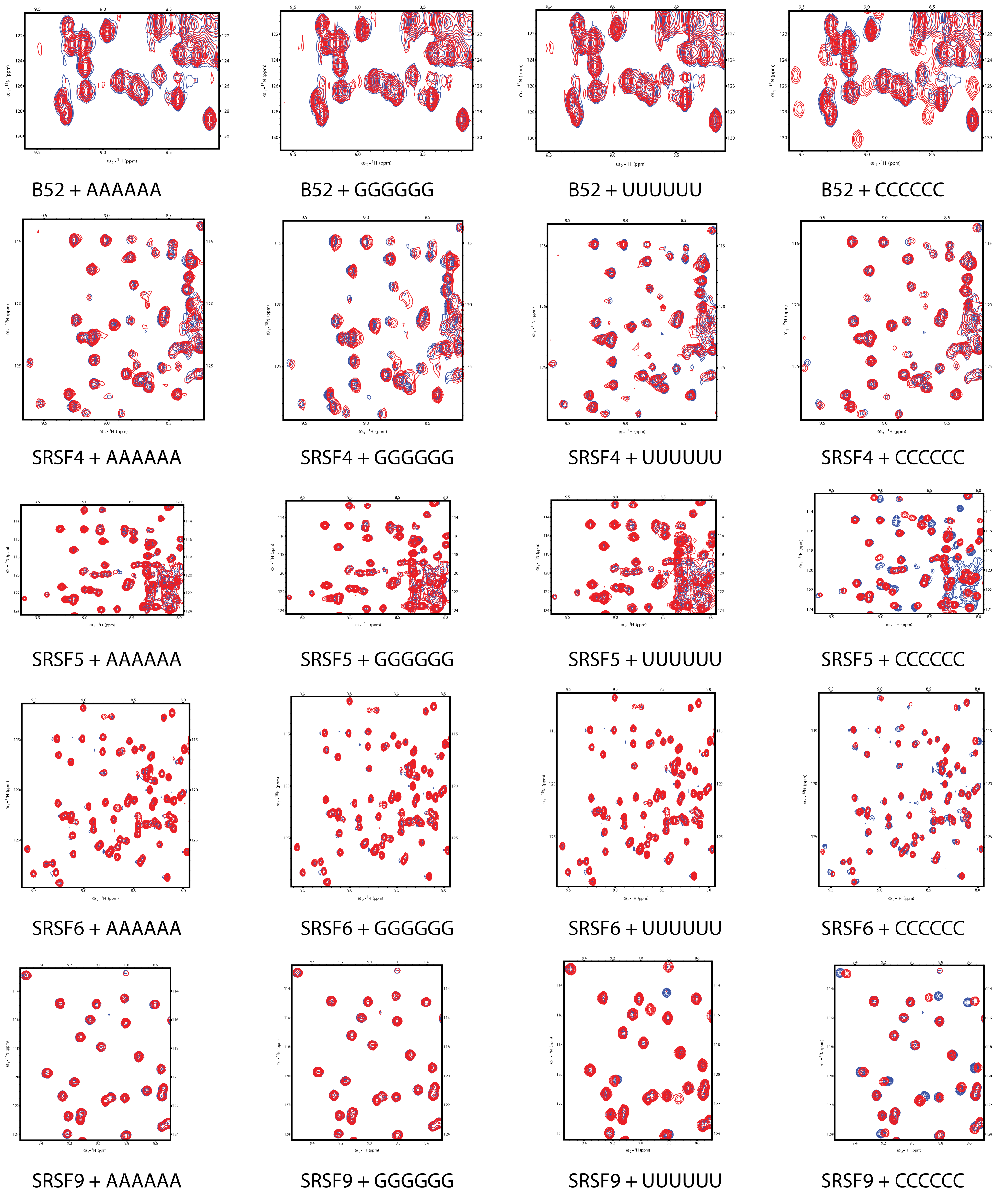

for all spectra: blue: protein free
red: protein:RNA=1:1

Figure S2: RRM1 domains of pseudo-RRM containing SR proteins bind preferentially to cytosines
Overlay of 1H-15N HSQC spectra obtained with Drosophila B52 RRM1 and human SRSF4, SRSF5, SRSF6, SRSF9 RRM1 in their free form (in blue) and in the presence of polyA, polyG, polyC or polyU molecules (in red).

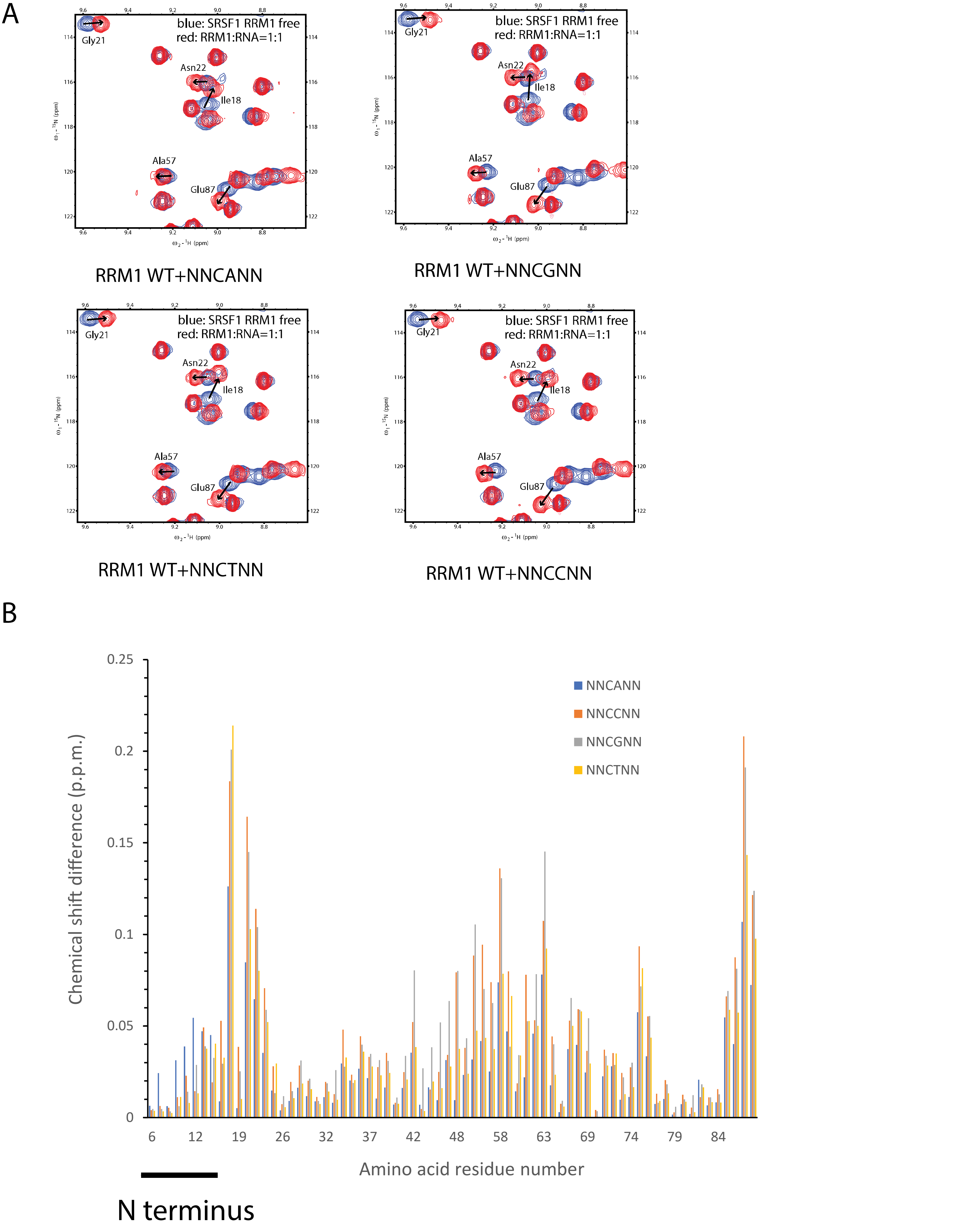

Figure S3: SRSF1 RRM1 binds to a CN motif (N is for A, C, G or U)
(A) Overlay of 1H-15N HSQC spectra measured with SRSF1 RRM1 free form (in blue) and in the presence of NNCANN, NNCGNN, NNCTNN and NNCCNN ssDNA (in red). **(B)** Mapping of the chemical shift perturbations observed upon interaction of SRSF1 RRM1 with these ssDNAs.

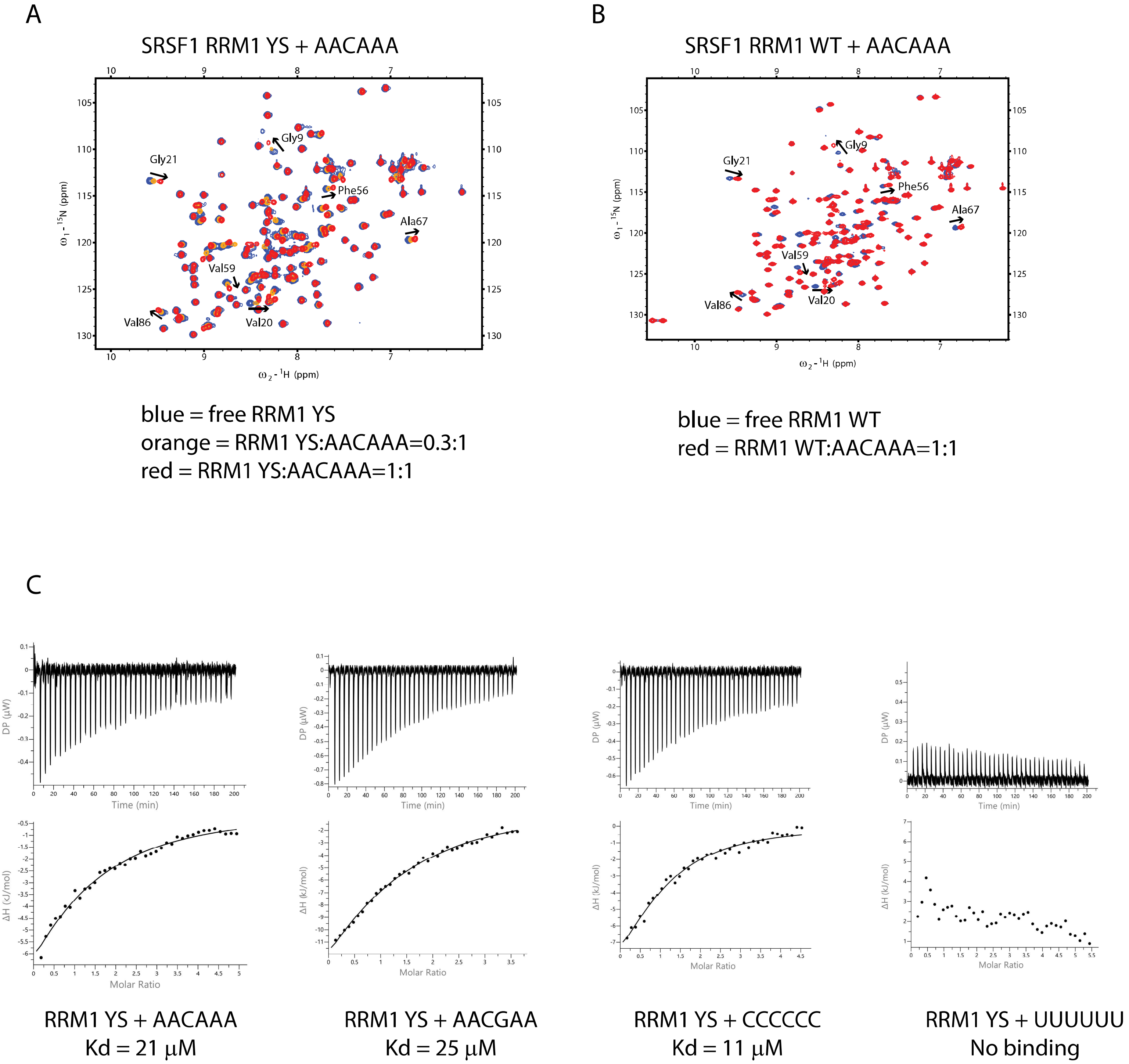

Figure S4: Investigation of SRSF1 RRM1 Y37S+Y72S and WT binding to RNA
(A) Overlay of 1H-15N HSQC spectra measured with SRSF1 RRM1 Y37S+Y72S (YS) free form (in blue) and bound to AACAAA RNA at a 0.3:1 and 1:1 RNA:protein ratio (in orange and red, respectively). **(B)** Overlay of 1H-15N HSQC spectra measured with SRSF1 RRM1 WT free form (in blue) and bound to AACAAA RNA at a 1:1 RNA:protein ratio (in red). Similar chemical shift perturbations are observed at saturation (at a RNA:protein ratio of 1:1) showing that the mode of interaction with RNA and the affinity is similar for the WT and Y37S+Y72S versions of SRSF1 RRM1. **(C)** ITC titrations performed at 40°C with SRSF1 RRM1 YS and AACAAA, AACGAA, CCCCCC and UUUUUU RNAs.

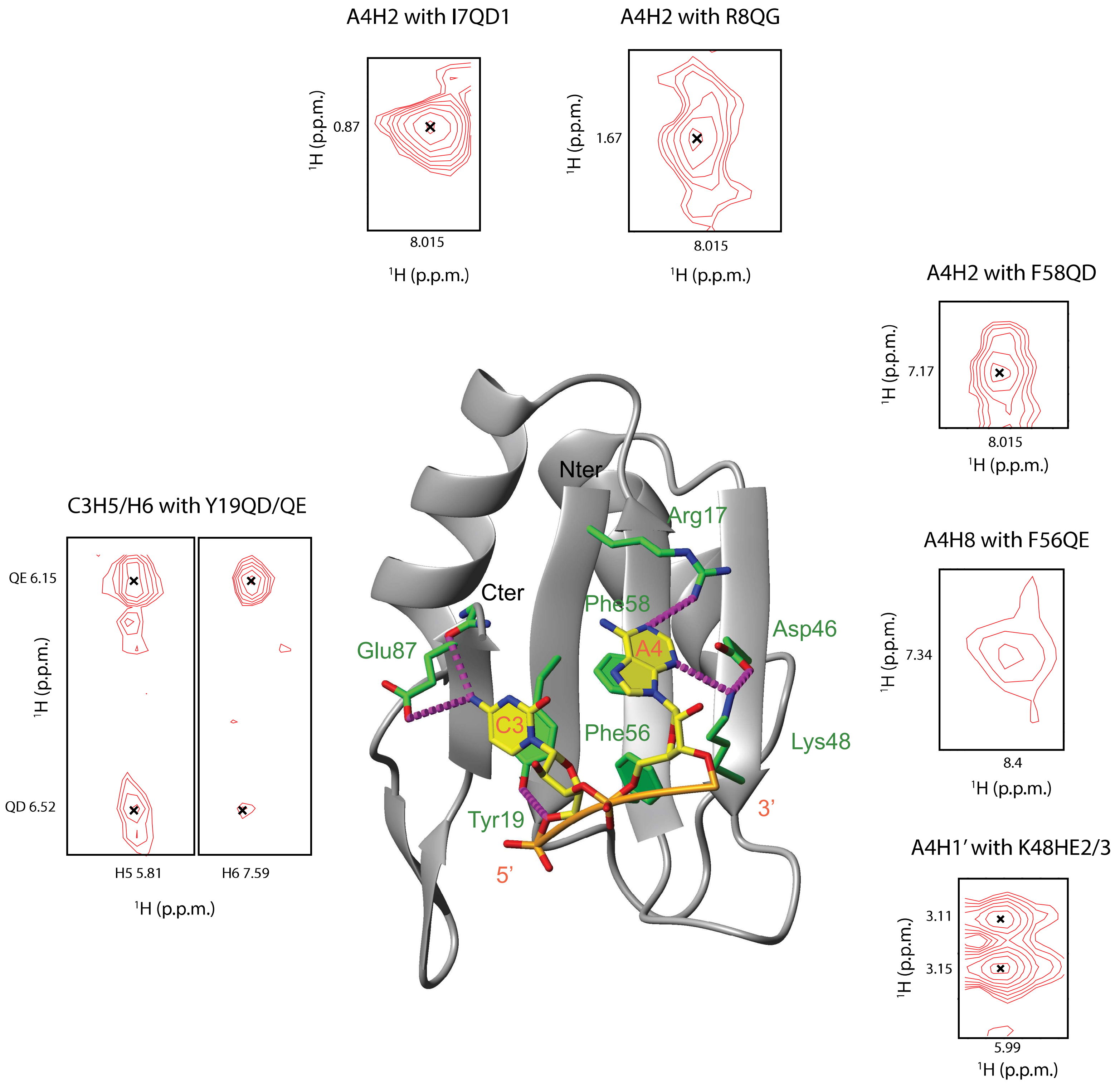

Figure S5: Presentation of most characteristic intermolecular NOEs observed between SRSF1 RRM1 and the 5'-AACAAA-3' RNA.
Representative intermolecular NOEs observed in 2D 1F-edited 2F-filtered NOESY experiments are shown.

A

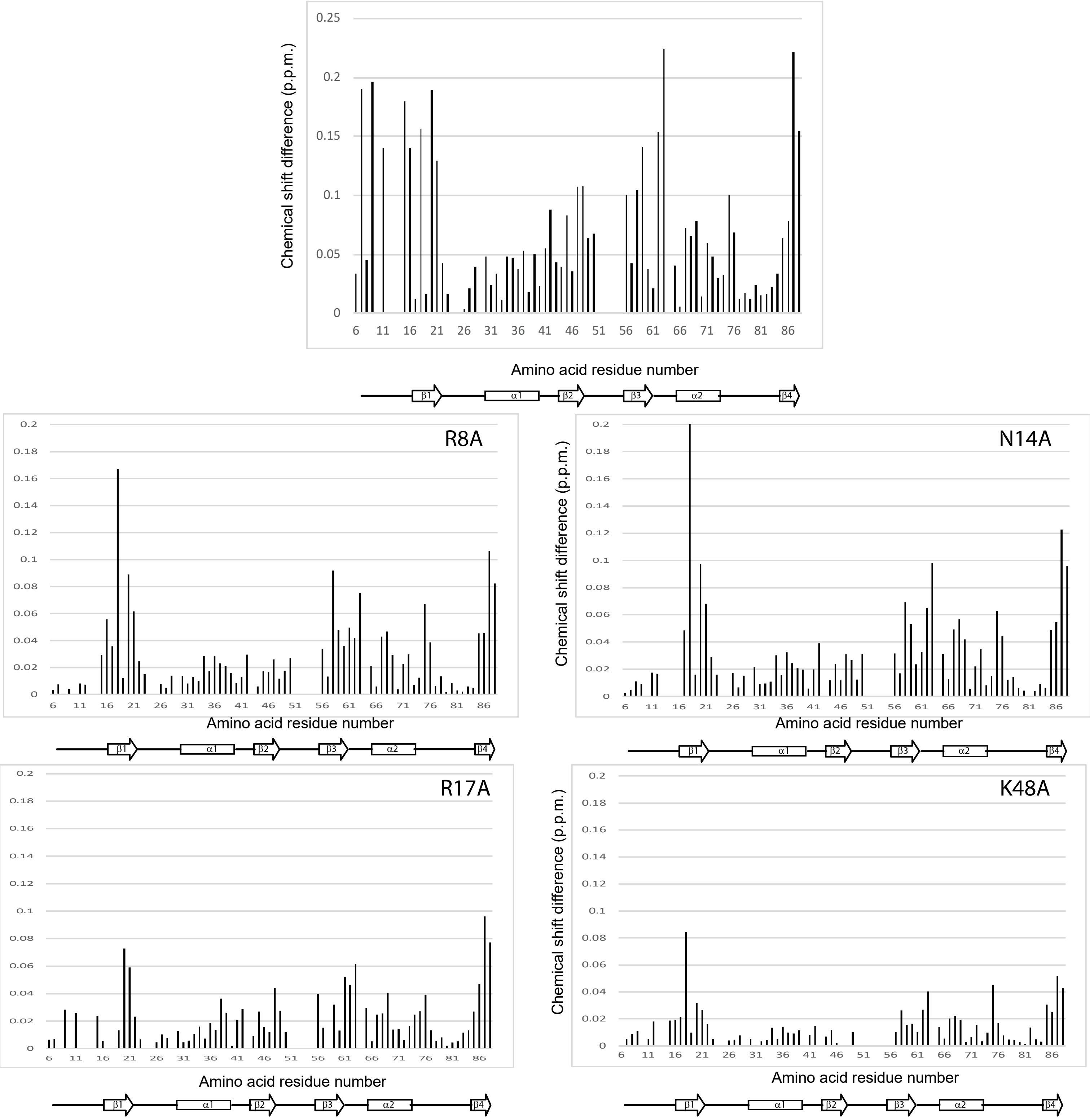

B

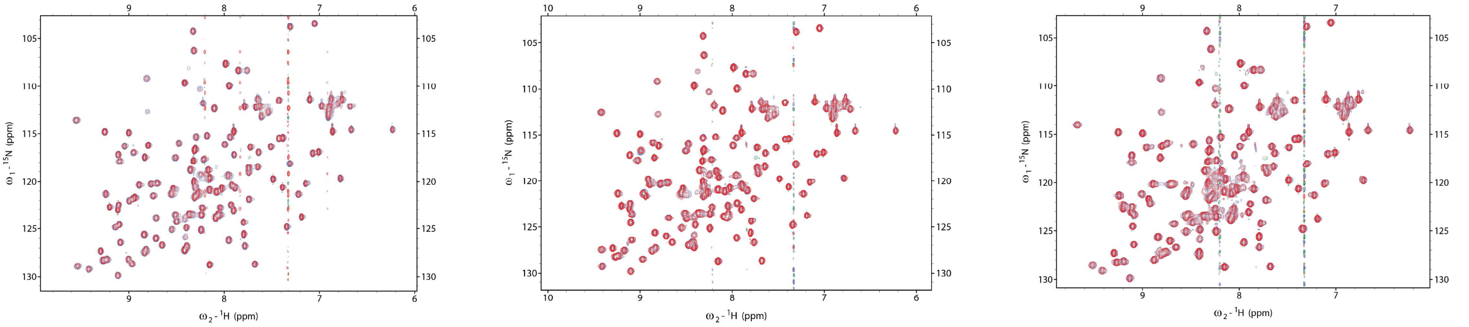

Y19A+AACAAA F56A+AACAAA F58A+AACAAA

Figure S6: Effect of R8A, N14A, R17A, Y19A, K48A, F56A and F58A on chemical shift perturbations observed upon binding of these SRSF1 RRM1 variants to AACAAA ssDNA.
(A) Representation of the combined chemical-shift perturbations of R8A, N14A, R17A and K48A SRSF1 variant amides upon binding to 5'-AACAAA-3' ssDNA, as a function of RRM1 amino acid sequence. Secondary-structure elements of the protein domain are displayed at the bottom of the graphs. **(B)** Superimposition of 1H-15N HSQC spectra recorded with 15N-labeled SRSF1 RRM1 free (in blue) and in the presence of AACAAA ssDNA (in red). No chemical shift perturbation was observed upon addition of ssDNA. All measurements were performed at 40°C (313K), in the NMR buffer.

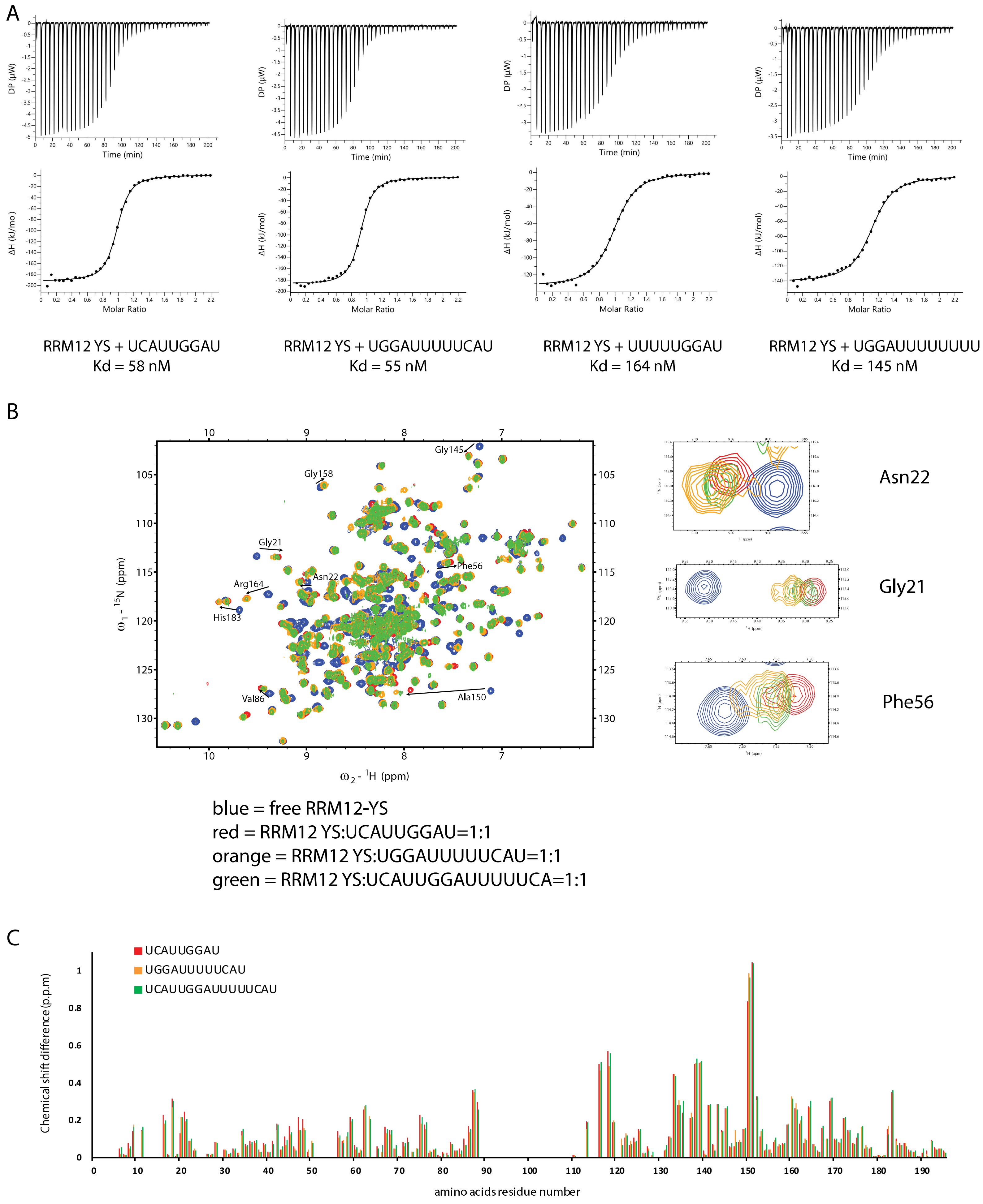

Figure S7: Investigation of SRSF1 RRM12 Y37S+Y72S binding to RNA
(A) ITC titrations performed at 40°C with SRSF1 RRM12 YS and UCAUUGGAU, UGGAUUUUUCAU, UUUUUGGAU and UGGAUUUUUUUU RNAs. **(B)** Overlay of 1H-15N HSQC spectra measured with SRSF1 RRM12 YS free form (in blue) and bound to UCAUUGGAU, UGGAUUUUUCAU and UCAUUGGAUUUUU-CAU RNAs at a 1:1 RNA:protein ratio (in red, orange and green, respectively). **(C)** Representation of the combined chemical-shift perturbations observed in the panel B.

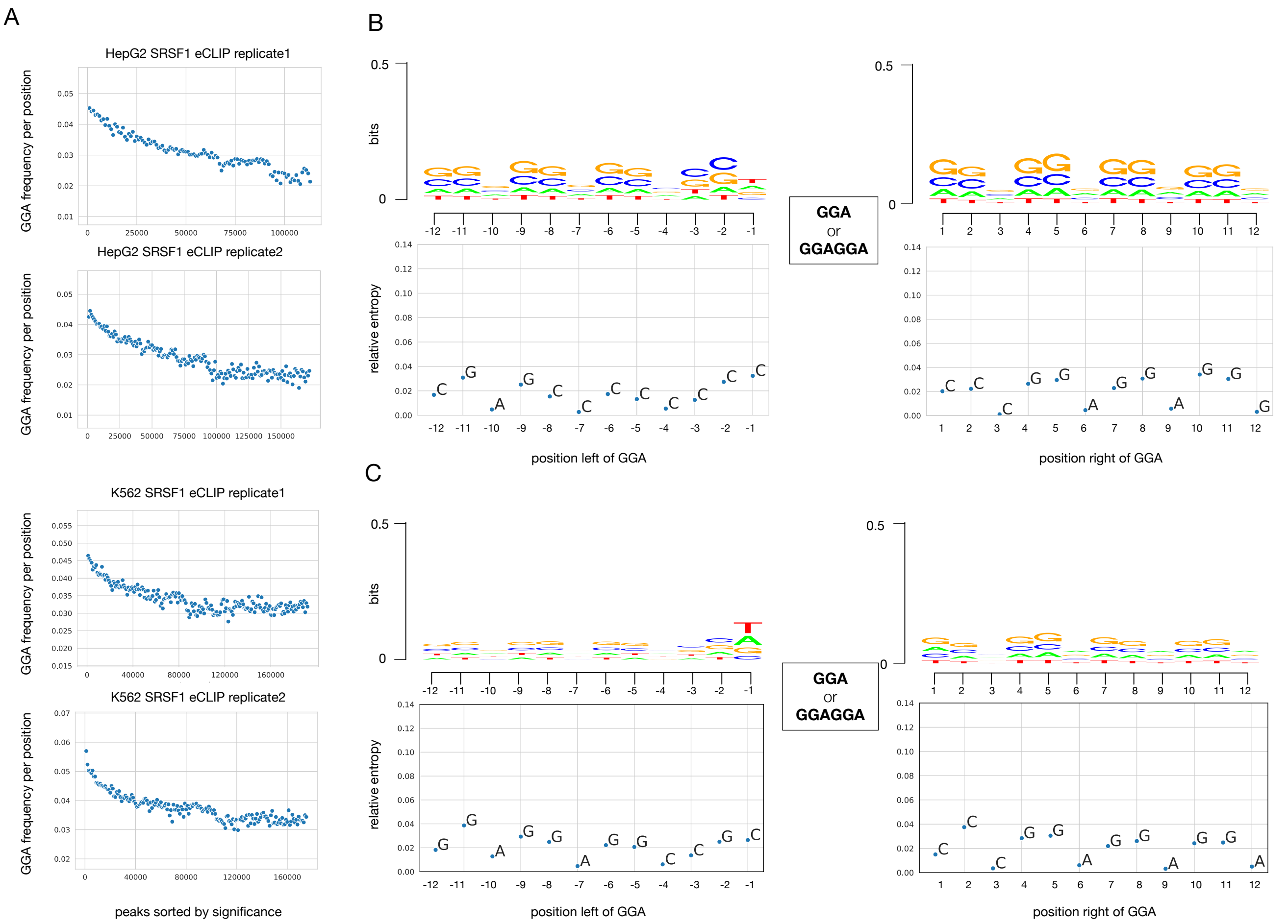

Figure S8: Re-analysis of eCLIP data of SRSF1 (obtained from ENCODE) in two human cell lines (HepG2 and K562). (A) Average frequency of GGA motifs per peak position for non-overlapping bins of 1000 peaks obtained from ENCODE and sorted from most to least significant (based on p-value). The frequency of GGAs decreases from the most significant to the least significant peaks, for both cell lines and all replicates. **(B)** Sequence logo of nucleotide frequencies and position-dependent relative entropy around the GGA motifs in SRSF1 eCLIP-derived peaks from human HepG2 cells. A weak enrichment for C is observed at multiple positions in the vicinity of the GGA motif. **(C)** The same observations are made for K562 cell line, with a lower signal strength.

Table S1: Structural statistics of the SRSF1 RRM1 in complex with AACAAA RNA

	Protein	RNA
**NMR distance and dihedral constraints**		
Distance restraints		
Total NOE	1537	6
Intra-residue	333	6
Inter-residue		
Sequential ($\|i - j\| = 1$)	416	0
Nonsequential ($\|i - j\| > 1$)	788	0
Hydrogen bonds	15	0
Protein–RNA intermolecular	33	
Total dihedral angle restraints		6
Protein		
ϕ	0	
ψ	0	
Nucleic acid		
Base pair		0
Sugar pucker		6
Backbone		0
Based on A-form geometry		0
**Structure statistics**		
Violations (mean and s.d.)		
Number of distance constraints (>0.3 Å) (Å)	1 ± 0.5	
Dihedral angle constraints (º)	0	
Max. dihedral angle violation (º)	0	
Max. distance constraint violation (Å)	0.33 ± 0.02	
Deviations from idealized geometry		
Bond lengths (Å)	0.0042 ± 0.0001	
Bond angles (º)	1.228 ± 0.016	
Average pairwise r.m.s. deviation** (Å)		
Protein		
Heavy	1.75 ± 0.24	
Backbone	1.15 ± 0.25	
RNA		
All RNA heavy		0.54 ± 0.14
Complex		
Protein and RNA heavy		1.71 ± 0.23

** Protein r.m.s. deviation was calculated using residues 17 to 88 for the ensemble of 20 refined structures. RNA r.m.s. deviation was calculated using nucleotides 3 and 4 for the ensemble of 20 refined structures.

Protein	Selected in vitro	Selected in vivo	Method	Reference
**SRSF1**				
RRM12	AGGA		RNAcompete	Ray et al. 2009 (25)
FL		GGAGA	CLIP-seq in mouse	Pandit et al., 2013 (23)
FL		AGGA	iCLIP in mouse	Müller-McNicoll et al., 2016 (39)
RRM12	RGAAGAAC		SELEX	Tacke and Manley 1995 (22)
RRM12	AGGACAGAGC		SELEX	Tacke and Manley 1995 (22)
RRM1	ACGCGCA		SELEX	Tacke and Manley 1995 (22)
FL		UCAGAGGA	RNA-seq	Anczuków et al. 2015 (31)
RRM2	GGA		NMR structure	Clery et al., 2013 (20)
FL		GAAGAAG	CLIP in human	Sanford et al., 2009 (28)
FL	SRSASGA		functional SELEX, R=A/G, S=C/G, W=A/U, M=A/C	Liu et al., 1998 (24)
FL	CRSMSGW		functional SELEX, R=A/G, S=C/G, W=A/U, M=A/C	Categni et al., 2002 (7)
FL	CcccGG/cA		functional SELEX	Smith et al., 2006 (33)
RRM12	GGAGGA		RNAcontext	Ray et al. 2013 (26)
FL		AGAAGAAG	CLIP in human	Wang et al., 2011 (29)
**SRSF5**				
RRM12	UGGGAGCRGUYRGCUCGY		SELEX	Tacke et al., 1997 (40)
FL	ACDGS		Functional SELEX	Liu et al., 1998 (24)
FL		UCGGA	CLIP in mouse	McNicoll et al., 2016 (39)
**SRSF6**				
RRM12	GAUCAACCUGGCGAC		SELEX	Shi et al., 1997 (27)
FL	USCGKM		Functional SELEX	Liu et al., 1998 (24)
FL		GAU$_{/A}$GA	CLIP in mouse	McNicoll et al., 2016 (39)

Table S2: Summary of the consensus sequences found *in vitro* and *in vivo* with SRSF1. Our structural studies allow the identification of the motifs recognized by SRSF1 RRM1 and RRM2, which are colored in green and red, respectively. R=A/G, Y=C/U, S=C/G, K=G/U, W=A/U, M=A/C, D=A/G/U.
