# Supplementary material for: Structure of SRSF1 RRM1 bound to RNA reveals an unexpected bimodal mode of interaction and explains its involvement in SMN1 exon7 splicing
Source: Nat Commun. 2021 Jan 18;12:428. doi: 10.1038/s41467-020-20481-w (PMC7813835; doi:10.1038/s41467-020-20481-w)
Supplement: Supplementary file 3 — Reporting Summary [file 41467_2020_20481_MOESM3_ESM.pdf]

nature research

Corresponding author(s): Frederic Allain & Antoine Clery

Last updated by author(s): Nov 23, 2020

Reporting Summary

Nature Research wishes to improve the reproducibility of the work that we publish. This form provides structure for consistency and transparency in reporting. For further information on Nature Research policies, see our Editorial Policies and the Editorial Policy Checklist.

Statistics

For all statistical analyses, confirm that the following items are present in the figure legend, table legend, main text, or Methods section.

n/a	Confirmed	
☐	☒	The exact sample size (*n*) for each experimental group/condition, given as a discrete number and unit of measurement
☐	☒	A statement on whether measurements were taken from distinct samples or whether the same sample was measured repeatedly
☐	☒	The statistical test(s) used AND whether they are one- or two-sided<br>*Only common tests should be described solely by name; describe more complex techniques in the Methods section.*
☒	☐	A description of all covariates tested
☒	☐	A description of any assumptions or corrections, such as tests of normality and adjustment for multiple comparisons
☐	☒	A full description of the statistical parameters including central tendency (e.g. means) or other basic estimates (e.g. regression coefficient) AND variation (e.g. standard deviation) or associated estimates of uncertainty (e.g. confidence intervals)
☐	☒	For null hypothesis testing, the test statistic (e.g. *F*, *t*, *r*) with confidence intervals, effect sizes, degrees of freedom and *P* value noted<br>*Give P values as exact values whenever suitable.*
☒	☐	For Bayesian analysis, information on the choice of priors and Markov chain Monte Carlo settings
☒	☐	For hierarchical and complex designs, identification of the appropriate level for tests and full reporting of outcomes
☒	☐	Estimates of effect sizes (e.g. Cohen's *d*, Pearson's *r*), indicating how they were calculated

Our web collection on statistics for biologists contains articles on many of the points above.

Software and code

Policy information about availability of computer code

Data collection	Topspin 3.6.2 (bruker)
Data analysis	CYANA 3.98.13, sparky 3.133, atnoscandid 2.1, AMBER12 (structure calculation),Molmol 2K.2, AMBER 16 (MD), VMD 1.9.2 (MD), origin 7.0383

For manuscripts utilizing custom algorithms or software that are central to the research but not yet described in published literature, software must be made available to editors and reviewers. We strongly encourage code deposition in a community repository (e.g. GitHub). See the Nature Research guidelines for submitting code & software for further information.

Data

Policy information about availability of data

All manuscripts must include a data availability statement. This statement should provide the following information, where applicable:

- Accession codes, unique identifiers, or web links for publicly available datasets
- A list of figures that have associated raw data
- A description of any restrictions on data availability

The chemical shifts and the atomic coordinates of the structure of SRSFl-RRMl bound to RNA have been deposited in the Protein Data Bank (PDB ID: 6HPJ). Other data and materials are available from the authors upon reasonable request.

April 2020

Field-specific reporting

Please select the one below that is the best fit for your research. If you are not sure, read the appropriate sections before making your selection.

☒ Life sciences ☐ Behavioural & social sciences ☐ Ecological, evolutionary & environmental sciences

For a reference copy of the document with all sections, see nature.com/documents/nr-reporting-summary-flat.pdf

Life sciences study design

All studies must disclose on these points even when the disclosure is negative.

Sample size	For all the biochemical assays, N=3. This sample size is in agreement with standard RT-PCR protocol: we transfected cells independently 3 times then performed RT-PCR on these 3 independent samples and performed statistics on these 3 independent set of data.
Data exclusions	No data exclusion.
Replication	3 biological replicates were performed for the splicing assays. All attempts were successful.
Randomization	The samples were not randomized (not relevant here).
Blinding	Blinding was not used in this study (not relevant here).

Reporting for specific materials, systems and methods

We require information from authors about some types of materials, experimental systems and methods used in many studies. Here, indicate whether each material, system or method listed is relevant to your study. If you are not sure if a list item applies to your research, read the appropriate section before selecting a response.

Materials & experimental systems

n/a	Involved in the study	
☐	☒	Antibodies
☐	☒	Eukaryotic cell lines
☒	☐	Palaeontology and archaeology
☒	☐	Animals and other organisms
☒	☐	Human research participants
☒	☐	Clinical data
☒	☐	Dual use research of concern

Methods

n/a	Involved in the study	
☒	☐	ChIP-seq
☒	☐	Flow cytometry
☒	☐	MRI-based neuroimaging

Antibodies

Antibodies used	ANTI-FLAG M2 Antibody (mouse) from sigma, horseradish-peroxidase-conjugated anti-mouse (Sigma)
Validation	Anti Flag M2 antibody is used for the detection of Flag fusion proteins.This monoclonal antibody is produced in mouse and recognizes the FLAG sequence at the N-terminus, Met N-terminus, and C-terminus. The antibody is also able to recognize FLAG at an internal site. M2, unlike M1 antibody is not Calcium dependent.<br>F1804 is an affinity purified, FLAG M2 antibody, increasing sensitivity in most applications.

Eukaryotic cell lines

Policy information about cell lines

Cell line source(s)	HEK293T cells obtained from the European collection of cell cultures (ECACC No. 85120602)
Authentication	None of the cell lines used were authentified.
Mycoplasma contamination	The cells were not tested for mycoplasma contaminations.
Commonly misidentified lines (See ICLAC register)	No commonly misidentified cell lines were used in this study.
