# Supplementary figures and images for: Structure of SRSF1 RRM1 bound to RNA reveals an unexpected bimodal mode of interaction and explains its involvement in SMN1 exon7 splicing
Source: Nat Commun. 2021 Jan 18;12:428. doi: 10.1038/s41467-020-20481-w (PMC7813835; doi:10.1038/s41467-020-20481-w)

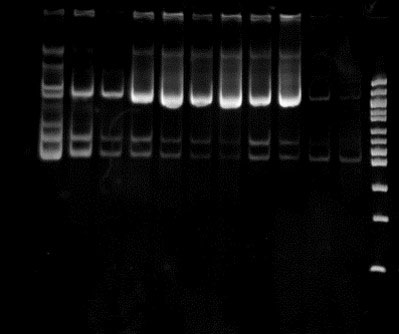

Supplement: Supplementary file 4 — Source data [file 41467_2020_20481_MOESM4_ESM.zip › source_data_file/fig3C_full_scan_gel_WT_and_ladder.jpg]

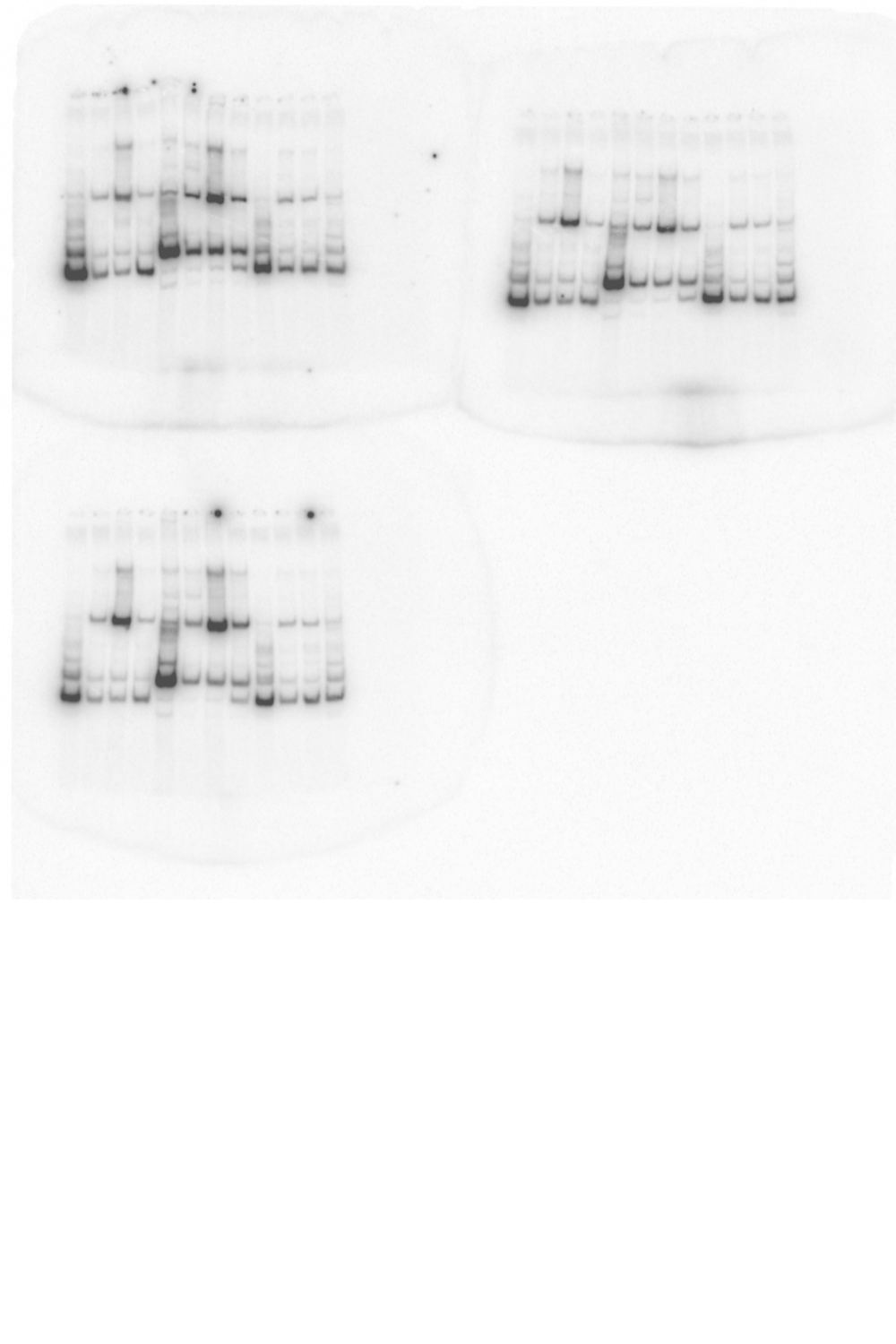

Supplement: Supplementary file 4 — Source data [file 41467_2020_20481_MOESM4_ESM.zip › source_data_file/fig3C_full_scan_RTpcr.tif]

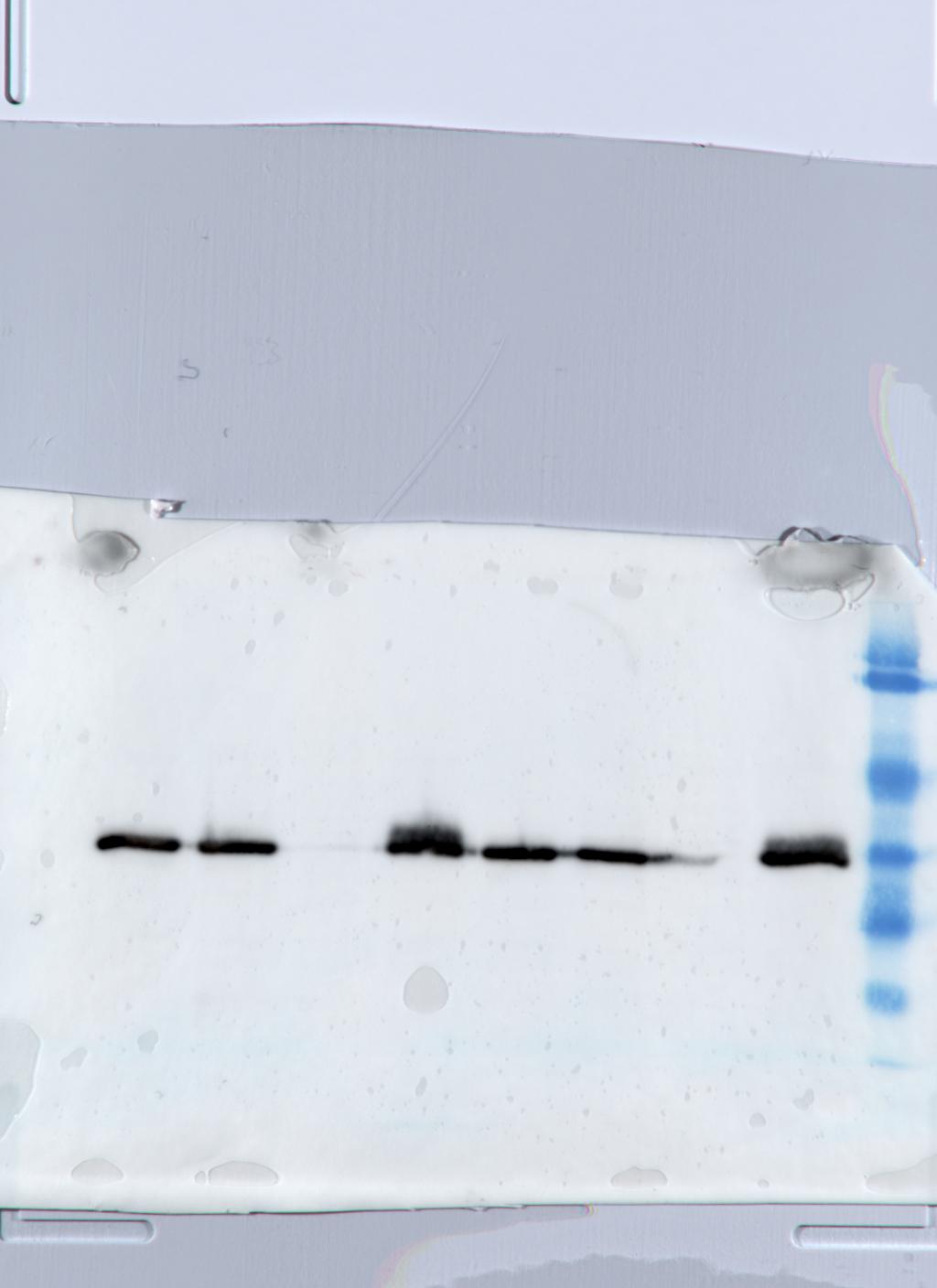

Supplement: Supplementary file 4 — Source data [file 41467_2020_20481_MOESM4_ESM.zip › source_data_file/fig3C_full_scan_WB.jpg]

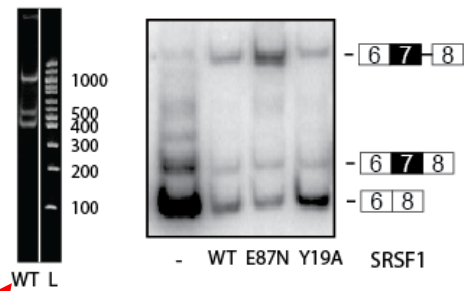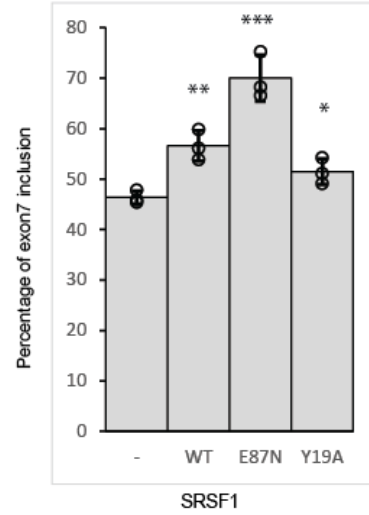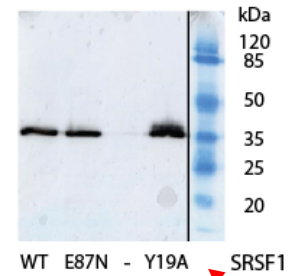

Figure 3

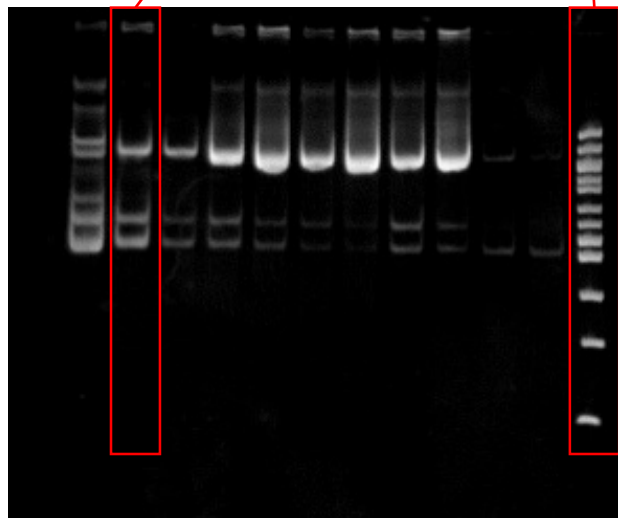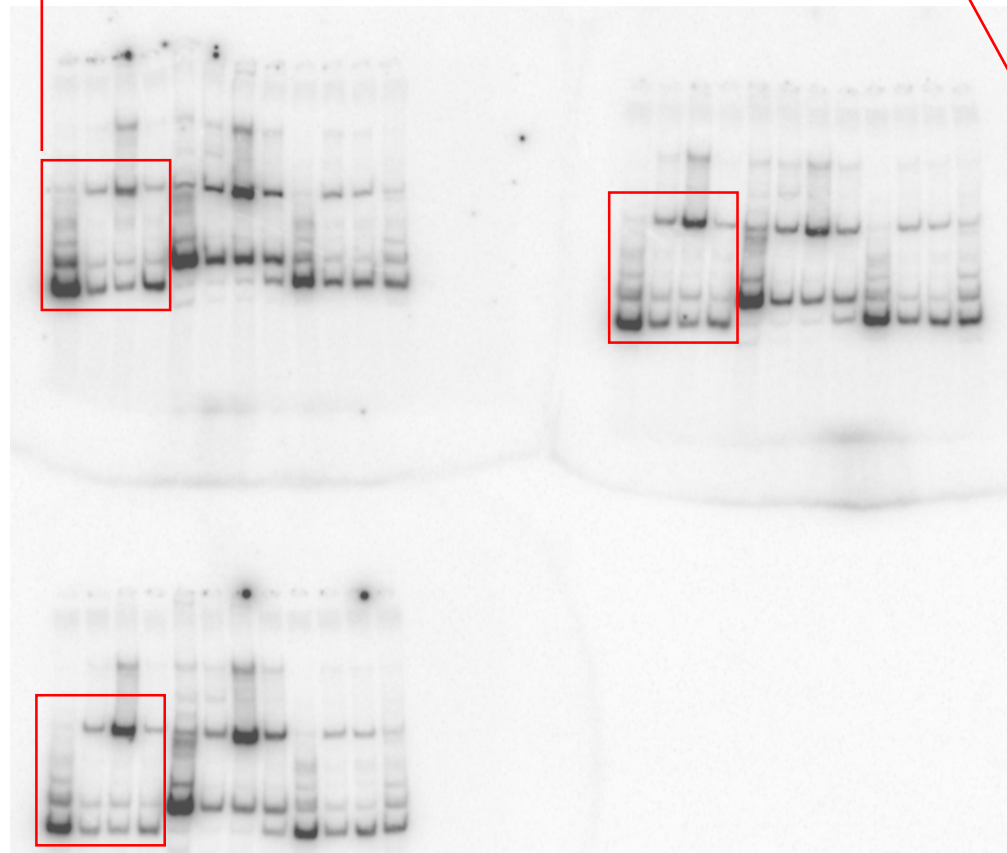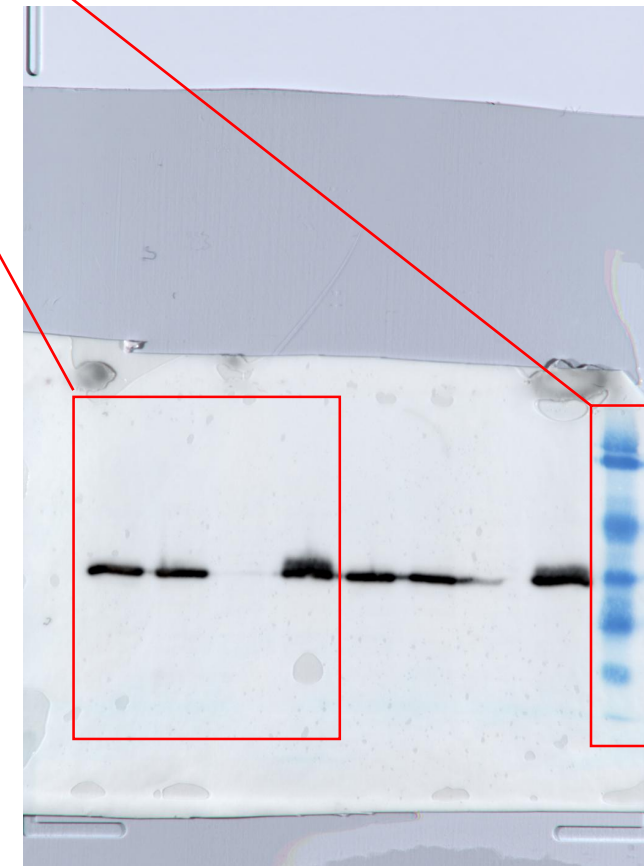

Supplement: Supplementary file 4 — Source data [file 41467_2020_20481_MOESM4_ESM.zip › source_data_file/Fig3C_raw_data.pdf]
